# Correction: Molecular modelling of the HCMV IL-10 protein isoforms and analysis of their interaction with the human IL-10 receptor

**DOI:** 10.1371/journal.pone.0295457

**Published:** 2023-11-30

**Authors:** Simone Queiroz Pantaleão, Lívia de Moraes Bomediano Camillo, Tainan Cerqueira Neves, Isabela de Godoy Menezes, Lucas Matheus Stangherlin, Helena Beatriz de Carvalho Ruthner Batista, Emma Poole, Michael Nevels, Eric Allison Philot, Ana Ligia Scott, Maria Cristina Carlan da Silva

The ninth author’s name is spelled incorrectly. The correct name is: Eric Allison Philot.

## References

[1] Pantaleão SQ, Camillo LMB, Neves TC, Menezes IG, Stangherlin LM, Batista HBCR, et al. (2022) Molecular modelling of the HCMV IL-10 protein isoforms and analysis of their interaction with the human IL-10 receptor. PloS ONE 17(11): e0277953. doi: 10.1371/journal.pone.0277953 36441804 PMC9704672

