# Supplementary figures and images for: Molecular modelling of the HCMV IL-10 protein isoforms and analysis of their interaction with the human IL-10 receptor
Source: PLoS One. 2022 Nov 28;17(11):e0277953. doi: 10.1371/journal.pone.0277953 (PMC9704672; doi:10.1371/journal.pone.0277953)

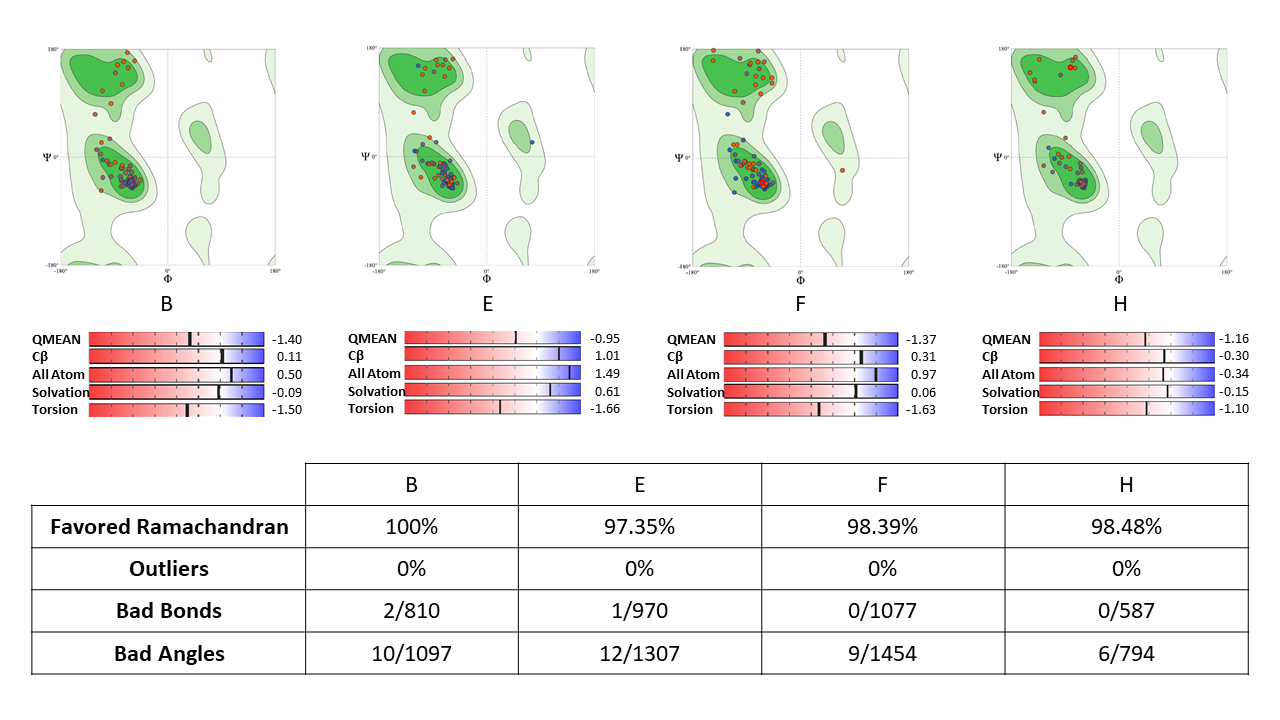

Supplement: S1 Fig — General case Ramachandran plot for the HCMV IL-10 isoforms B, E, F and H. (TIFF) [file pone.0277953.s001.tiff]

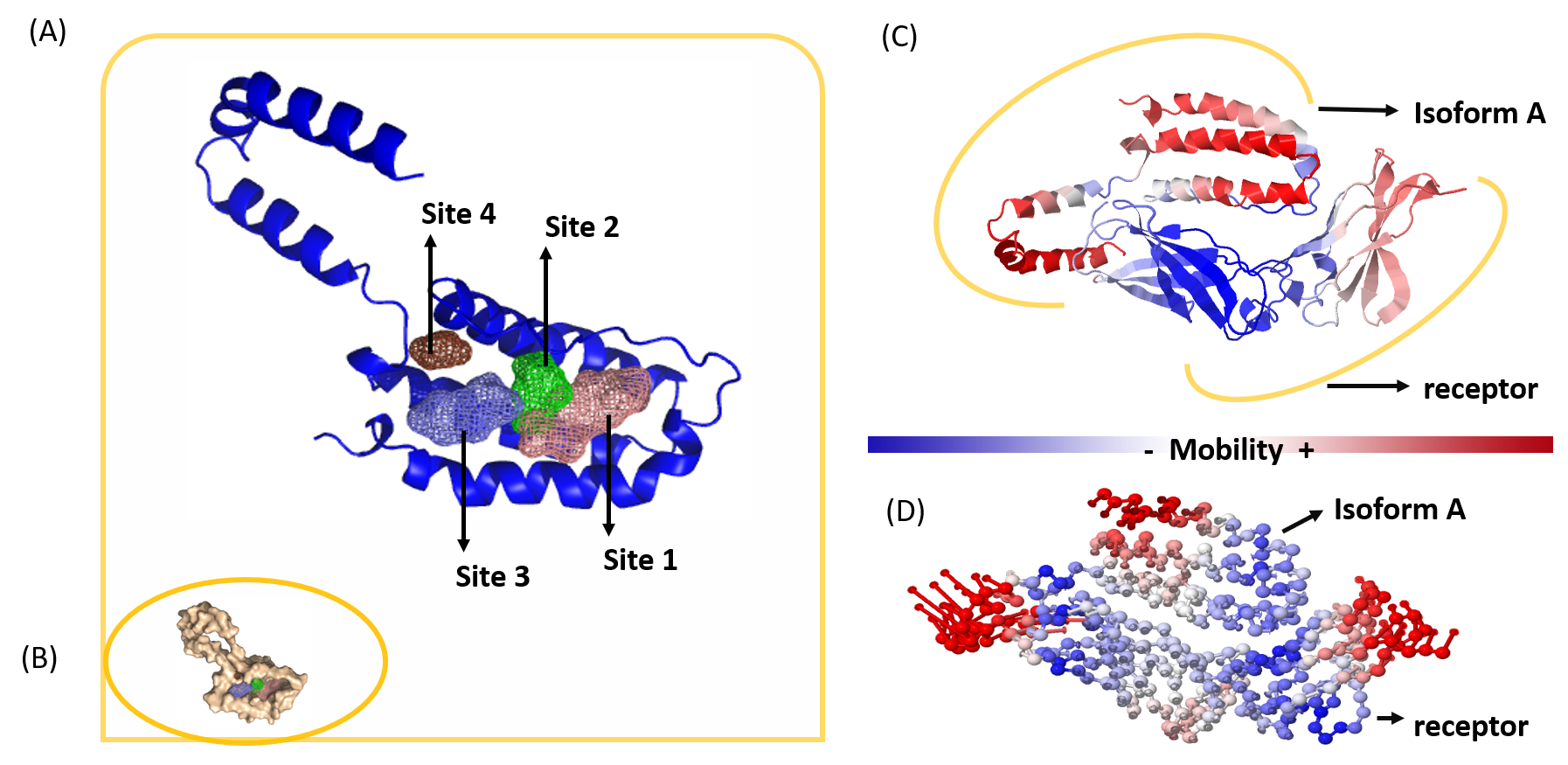

Supplement: S2 Fig — (A) Binding sites detected in the isoform. Site 1: Pink; site 2: Green; site 3: Lilac; site 4: Brown. (B) Representation of the isoforms on the surface. (C) Bioactive conformation of the isoforms coupled to the receptor. (D) Vectors obtained by calculating the normal modes, where we observe regions with less movement (more rigid) (blue), regions composed of residues with high movement (pink and red), and regions with intermediate movement (white). (TIF) [file pone.0277953.s002.tif]

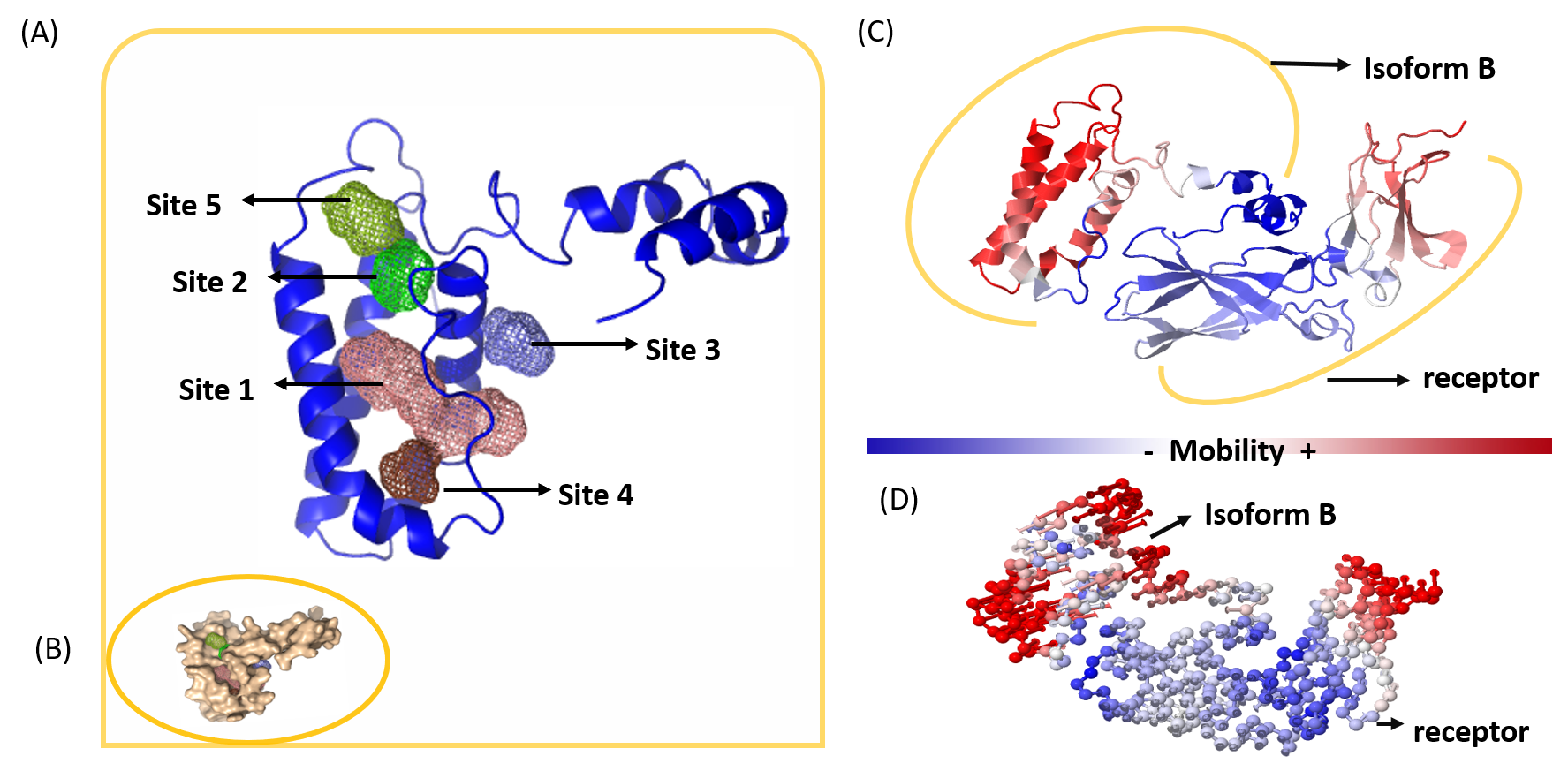

Supplement: S3 Fig — (A) Binding sites detected in the isoform. Site 1: Pink; site 2: Green; site 3: Lilac; site 4: Brown; site 5: Olive green. (B) Representation of the isoforms on the surface. (C) Bioactive conformation of the isoforms coupled to the receptor. (D) Vectors obtained by calculating the normal modes, where we observe regions with less movement (more rigid) (blue), regions composed of residues with high movement (pink and red), and regions with intermediate movement (white). (TIF) [file pone.0277953.s003.tif]

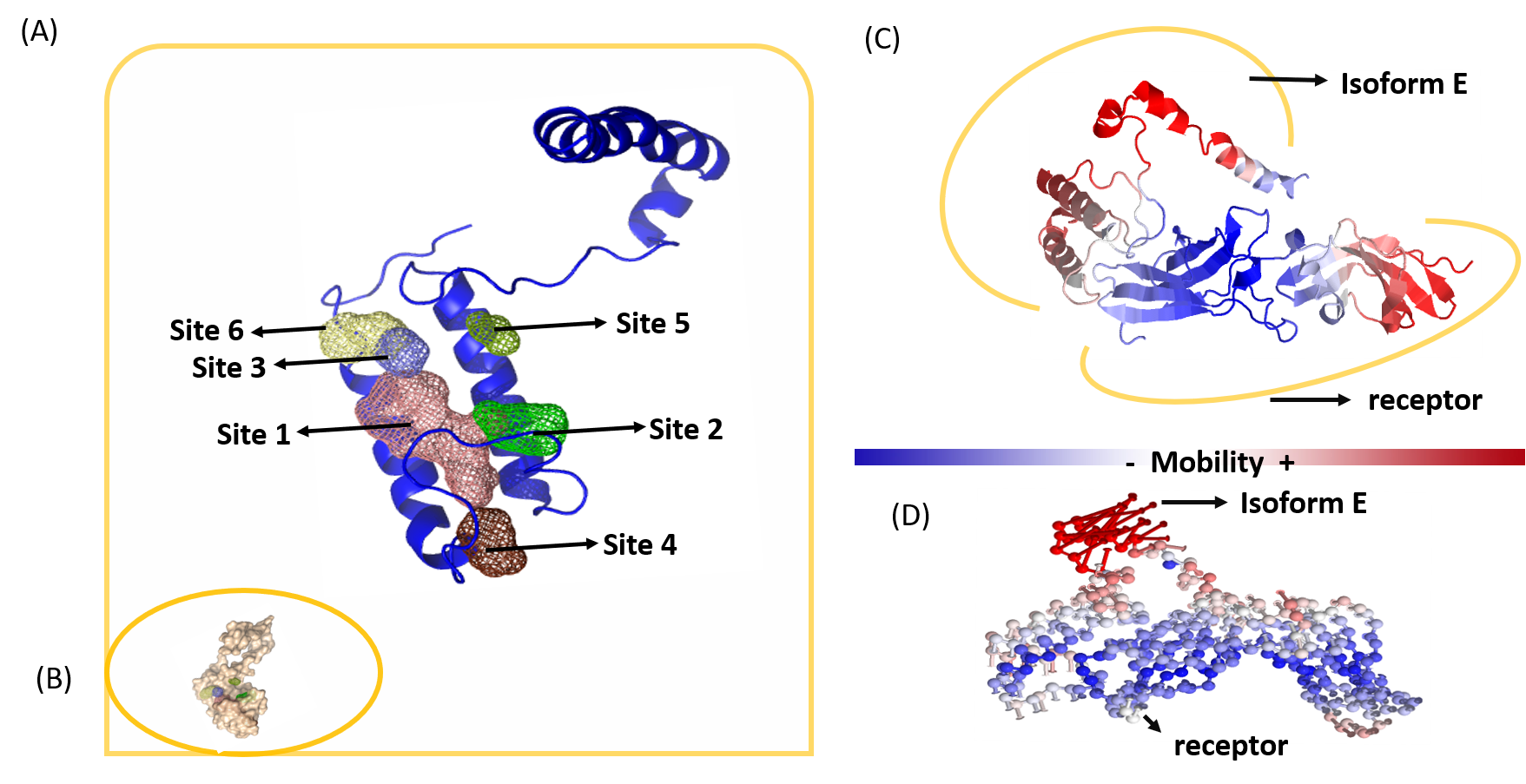

Supplement: S4 Fig — (A) Binding sites detected in the isoform. Site 1: Pink; site 2: Green; site 3: Lilac; site 4: Brown; site 5: Olive green; site 6: Grey. (B) Representation of the isoforms on the surface. (C) Bioactive conformation of the isoforms coupled to the receptor. (D) Vectors obtained by calculating the normal modes, where we observe regions with less movement (more rigid) (blue), regions composed of residues with high movement (pink and red), and regions with intermediate movement (white). (TIF) [file pone.0277953.s004.tif]

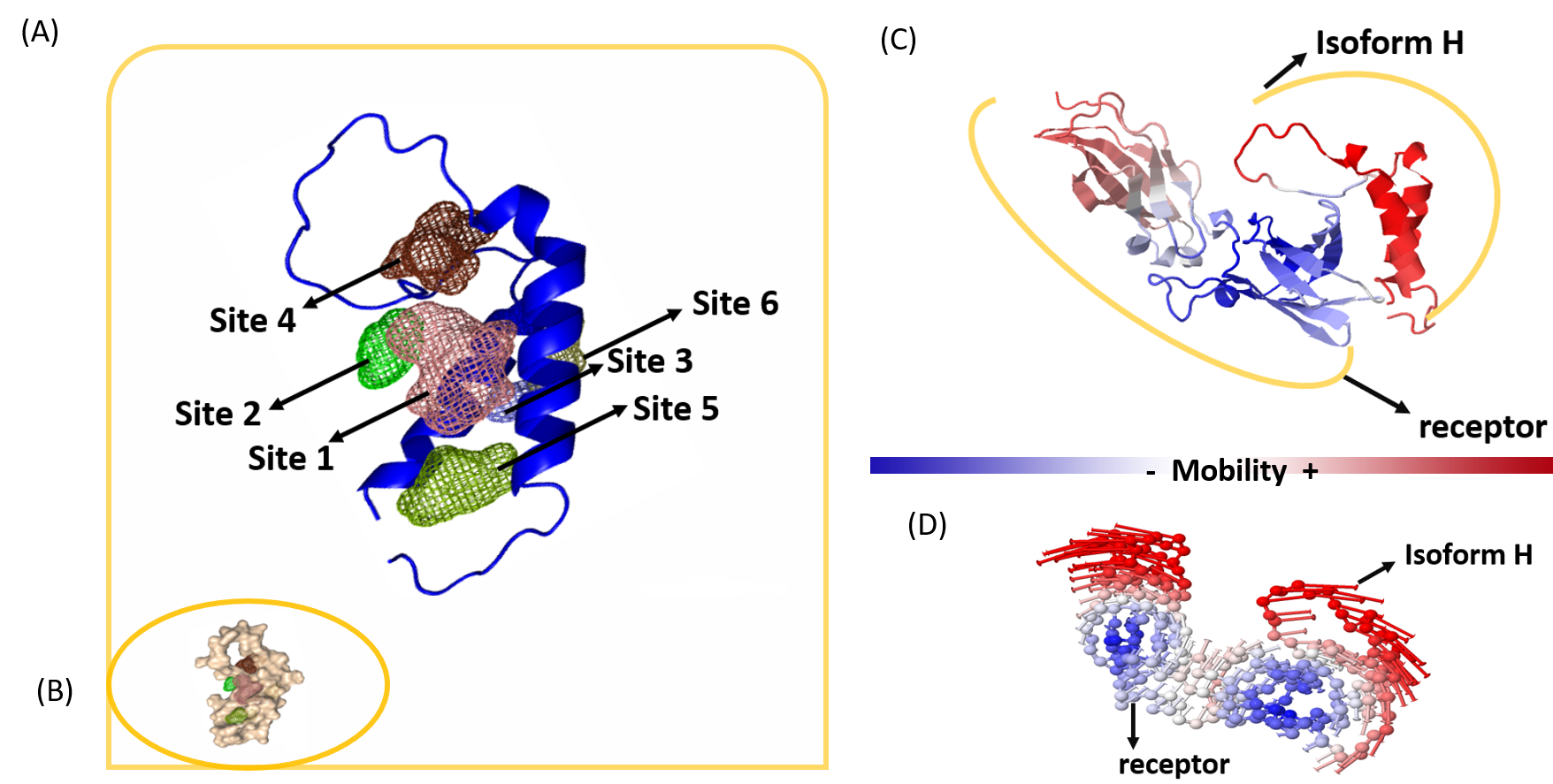

Supplement: S5 Fig — (A) Binding sites detected in the isoform. Site 1: Pink; site 2: Green; site 3: Lilac; site 4: Brown. (B) Representation of the isoforms on the surface. (C) Bioactive conformation of the isoforms coupled to the receptor. (D) Vectors obtained by calculating the normal modes, where we observe regions with less movement (more rigid) (blue), regions composed of residues with high movement (pink and red), and regions with intermediate movement (white). (TIF) [file pone.0277953.s005.tif]

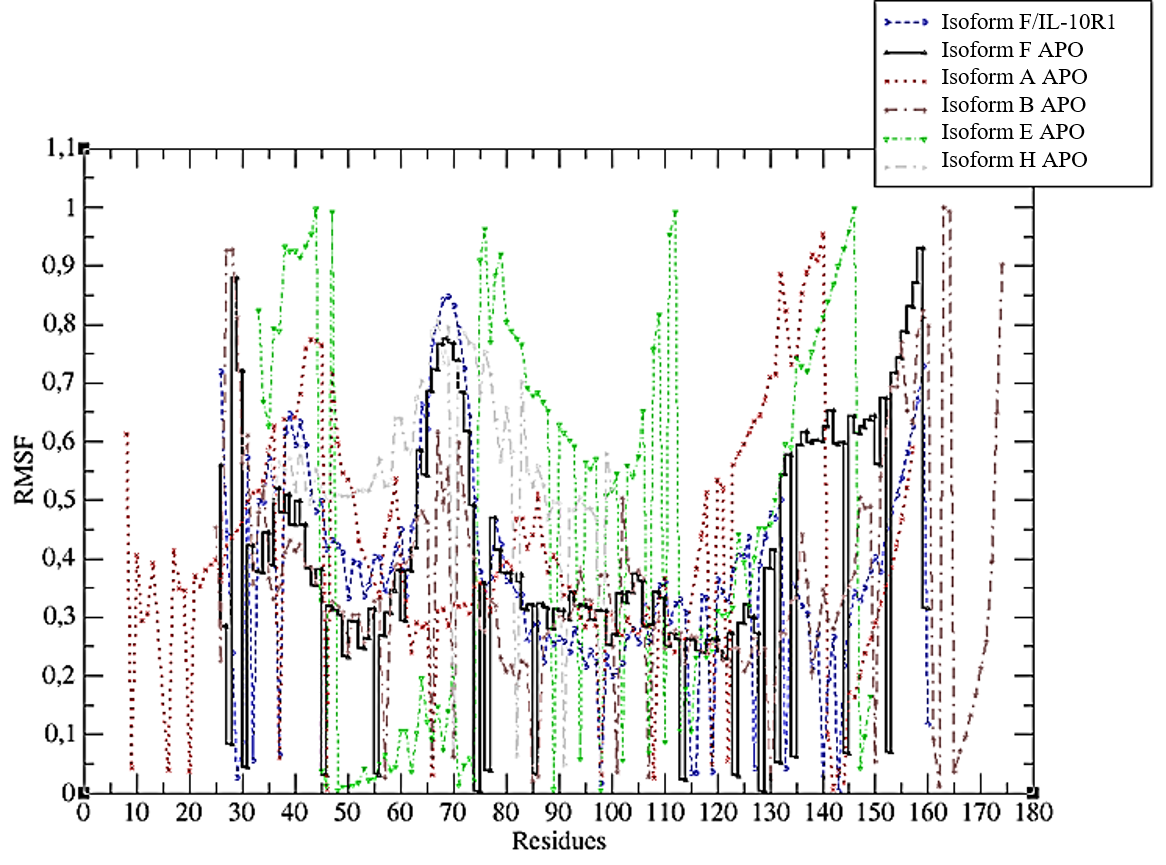

Supplement: S6 Fig — (TIF) [file pone.0277953.s006.tif]
