# Supplementary material for: Molecular modelling of the HCMV IL-10 protein isoforms and analysis of their interaction with the human IL-10 receptor
Source: PLoS One. 2022 Nov 28;17(11):e0277953. doi: 10.1371/journal.pone.0277953 (PMC9704672; doi:10.1371/journal.pone.0277953)
Supplement: S1 Table — (A) Description of amino acid residues detected in each binding site of the studied proteins. (B) Sizing of the binding sites found. (DOCX) [file pone.0277953.s007.docx]

**Table S1.**

1. **Description of amino acid residues detected in each binding site of the studied proteins***

| **Site** | **Isoform A** | **Isoform B** | **Isoform E** | **Isoform F** | **Isoform H** | **Receptor** |
| --- | --- | --- | --- | --- | --- | --- |
| **1** | LEU 23; LEU 26;  ARG 27; PHE 30;  LEU 37; TYR 72,  VAL 76; PHE 77;  GLY 80; TYR 84;  LEU 91; MET 94;  LEU 98 | LEU 47; LEU 50;  ARG 51; PHE 54;  LYS 58; VAL 69;  TRP 70; LEU 71;  ASP 72; GLY 73;  VAL 76; MET 85;  TRP 87; LEU 88;  ARG 91; TYR 92;  MET 114; LEU 118 | LEU 47; LEU 50;  ARG 51; PHE 54;  LEU 61; GLN 62;  TYR 67; GLU 72;  VAL 76; TYR 77;  LEU 80; LEU 84;  MET 87; ARG 88;  LEU 91; TYR 95 | LEU 50; ARG 51;  PHE 54; HIS 55;  LEU 71; ASP 72;  GLY 73; THR 74;  MET 75; LYS 77;  VAL 84; TRP 87;  LEU 88; TYR 92 | LEU 47; LEU 50;  ARG 51; PHE54;  HIS 55; VAL 76;  MET 87; LEU 91;  ILE 94; TYR 95;  MET 98 | GLU 16; PHE 17;  PHE 18; HIS 19;  THR 105; VAL 106;  GLY 107; PRO 125;  PRO 127; |
| **2** | LEU 23; LEU 26;  LEU 48; MET 65;  LEU 68; LEU 69;  TYR 72; LEU 98 | LEU 47; VAL 76;  LYS 77; GLY 81;  CYS 82; MET 85;  MET 125; SER 130;  VAL 131 | LEU 47; LEU 50;  ARG 51; PHE 54;  LEU 61; GLN 62;  TYR 67; GLU 72;  VAL 76; TYR 77;  LEU 80; LEU 84;  MET 87; ARG 88;  LEU 91; TYR 95 | PRO 36; MET 125;  ARG 126; GLN 127;  TRP 128; GLU 129;  GLU 131; ARG 132 | HIS 75; VAL 76;  TYR 77; TYR 95 | LEU 40; LEU 41;  ARG 42; ILE 45;  SER 47; TRP 48;  ASN 49; VAL 65;  THR 66; LEU 67;  ASP 68; TYR 75 |
| **3** | PRO 16; TYR 19;  LEU 23; MET 65;  LEU 69; LEU 98;  ILE 101; MET 105;  LEU 111 | TRP 70; LEU 71;  TRP 80; SER 83;  VAL 84; TRP 87 | LEU 47; TYR 95;  MET 98 | CYS 82; SER 83;  ASP 86; MET 125 | ARG 46, LEU 50;  THR 90; LEU 91;  SER 93; ILE 94 | LEU 104; THR 105;  VAL 106; VAL 184;  PRO 186; MET 196;  TRP 197; SER 198;  LYS 199 |
| **4** | CYS 62; MET 65;  ASP 66; LEU 69;  TYR 102; MET 105;  ARG 106; LEU 111;  GLY 112 | PHE 54; VAL 57;  LYS 58; LEU 61;  GLN 62; ASP 65;  VAL 69; VAL 96 | LEU 61; GLN 62;  ARG 63; GLU 64;  ASP 65; ASP 66;  LEU 80 | TRP 80; SER 83;  VAL 84; TRP 87;  ARG 90; LYS 140;  GLY 141; GLU 144 | PHE 54; LYS 58;  THR 61; LEU 62;  GLN 63; ARG64;  HIS 75; VAL 76;  TYR 77; PRO 78;  LEU 84 | PHE 13; GLU 14;  ALA 15; PHE 97;  GLU 101; VAL 102;  THR 103; LYS 194 |
| **5** | **-** | GLN 37; CYS 38;  ARG 39; TYR 43;  MET 125; CYS 128;  SER 130 | TYR 95; LYS 96;  ARG 99; LEU 104 | ARG 91; TYR 92;  ILE 95; VAL 96 | LYS 35; PRO 36;  TYR 43; ARG 46;  LEU 47; ILE 94;  ASP 97; MET 98;  GLN 100 | TRP 12; PHE 13;  THR 93; ASN 94;  THR 95; ARG 96;  PHE 97; GLU 101 |
| **6** | **-** | **-** | GLU 41; ASP 42;  TYR 43; ALA 44;  LEU 47; MET 98;  LEU 103 | ASP 86; LEU 89;  TYR 122; MET 126;  ARG 127 | ASP 49; LEU 50;  THR 53; SER 86;  MET 87; THR 90 | ILE 118; ILE 150;  LEU 171; LEU 172;  THR 173; SER 174;  GLU 176; PHE 180; |

*The amino acid residues highlighted in red have already had their importance recognized in the literature, the other residues were characterized primarily by work

**(B). Sizing of the binding sites found**

|  | **Site** | **Isoform A** | **Isoform B** | **Isoform E** | **Isoform F** | **Isoform H** | **receptor** |
| --- | --- | --- | --- | --- | --- | --- | --- |
| **Volume Å^3^** | **1** | 1823.0 | 2553.0 | 2324.0 | 1862.0 | 1570.0 | 1044.0 |
|  | **2** | 1097.0 | 1008.0 | 914.6 | 1097.0 | 525.4 | 1584.0 |
|  | **3** | 1236.0 | 969.60 | 571.4 | 408.3 | 742.8 | 1115.0 |
|  | **4** | 1137.0 | 979.40 | 1002.0 | 1060.0 | 1446.0 | 983.2 |
|  | **5** | - | 845.3 | 614.2 | 545.2 | 1189.0 | 1061.0 |
|  | **6** | - | - | 857.5 | 705.4 | 651.5 | 1003.0 |
|  |  | | | | | | |
| **Area Å^2^** | **1** | 1435.0 | 1965.0 | 1845.0 | 1555.0 | 1230.0 | 804.7 |
|  | **2** | 954.0 | 918.9 | 785.3 | 922.5 | 523.5 | 1127.0 |
|  | **3** | 1050.0 | 756.7 | 492.0 | 418.7 | 610.5 | 845.3 |
|  | **4** | 930.7 | 891.9 | 864.8 | 917.6 | 1245.0 | 808.4 |
|  | **5** | - | 787.2 | 509.4 | 477.4 | 1049.0 | 896.8 |
|  | **6** | - | - | 724.5 | 602.6 | 571.0 | 844.9 |
