# Supplementary material for: Molecular modelling of the HCMV IL-10 protein isoforms and analysis of their interaction with the human IL-10 receptor
Source: PLoS One. 2022 Nov 28;17(11):e0277953. doi: 10.1371/journal.pone.0277953 (PMC9704672; doi:10.1371/journal.pone.0277953)
Supplement: S2 Table — (DOCX) [file pone.0277953.s008.docx]

**Table S2.**

Main molecular interactions detected by BINANA for each receptor-coupled isoform

| **Isoform** | **HB** | **HC** | **SB** | **T-shaped** |
| --- | --- | --- | --- | --- |
| **A** | *GLU(41):OE2(535) **SER(59):HG(909) **SER(59):OG(907)  *ASP(43):OD2(559) **HIS(19):HE2(278) **HIS(19):NE2(276)  *SER(63):OG(806) *SER(63):HG(808) **GLU(179):OE2(2833)  *ASP(66):OD2(852) **ARG(151):2HH2(2386) **ARG(151):NH2(2380)  *TRP(67):NE1(869) **GLU(200):HN(3165) **GLU(200):N(3151)  *ARG(71):NH1(956) *ARG(71):1HH1(960) **TRP(197):O(3098)  *ASP(117):OD2(1650) **GLU(179):HN(2834) **GLU(179):N(2820)  *TYR(153):OH(2230) *TYR(153):HH(2232) **SER(204):O(3215) | *ARG(40):CD(509) **LEU(58):CB(885)  *GLU(41):CD(533) **LE(21):CG2(307)  *GLU(41):CD(533) **ILE(21):CD1(311)  *GLU(41):CD(533) **SER(59):CB(904)  *TRP(67):CG(862) **GLU(200):CB(3156)  *TRP(67):CD1(863) **GLU(200):CB(3156)  *TRP(67):CE2(866) **LYS(199):CA(3130)  *TRP(67):CZ2(870) **LYS(199):CA(3130)  *TRP(67):CZ3(872) **LYS(199):CG(3137)  *TRP(67):CH2(874) **LYS(199):CA(3130)  *TRP(67):CH2(874) **LYS(199):CB(3134)  *TRP(67):CH2(874) **LYS(199):CG(3137)  *ARG(70):CD(927) **PHE(157):CE1(2476)  *ARG(70):CZ(931) **PHE(157):CD1(2472)  *VAL(83):CG2(1146) **TRP(12):CZ2(153)  *ARG(106):CZ(1528) **PHE(157):CZ(2480)  *ASP(117):CB(1645) **GLN(208):CB(3285)  *LYS(118):CB(1657) **PRÓ(154):CB(2430)  *ILE(121):CG1(1708) **VAL(177):CG2(2808)  *ILE(121):CG2(1711) **VAL(177):CG2(2808)  *ILE(121):CD1(1715) **PRÓ(154):CB(2430)  *ILE(121):CD1(1715) **PRÓ(154):CG(2433)  *ILE(121):CD1(1715) **GLY(178):CA(2814  *LEU(124):CD1(1765) **VAL(177):CG2(2808)  *LEU(154):CD2(2247) *HIS(114):CA(1757)  *LEU(154):CD2(2247) **HIS(114):C(1759) | [A:ASP(43):CG(557) / A:ASP(43):OD1(558) / A:ASP(43):OD2(559)] /  [B:HIS(19):CG(269) / B:HIS(19):CD2(270) / B:HIS(19):ND1(272) / B:HIS(19):HD1(273) / B:HIS(19):CE1(274) / B:HIS(19):NE2(276) / B:HIS(19):HE2(278)]  [A:GLU(41):CD(533) / A:GLU(41):OE1(534) / A:GLU(41):OE2(535)] /  [B:HIS(23):CG(343) / B:HIS(23):CD2(344) / B:HIS(23):ND1(346) / B:HIS(23):HD1(347) / B:HIS(23):CE1(348) / B:HIS(23):NE2(350) / B:HIS(23):HE2(352)]  [A:ASP(66):CG(850) / A:ASP(66):OD1(851) / A:ASP(66):OD2(852)] /  [B:ARG(151):CZ(2378) / B:ARG(151):NH1(2379) / B:ARG(151):NH2(2380) / B:ARG(151):1HH1(2383) / B:ARG(151):2HH1(2384) / B:ARG(151):1HH2(2385) / B:ARG(151):2HH2(2386)] | - |
| **B** | *TYR(67):OH(716) *TYR(67):HH(718) **GLU(176):OE1(2794)  *:TRP(70):NE1(761) **LYS(152):HZ3(2408) **:LYS(152):NZ(2404)  *TRP(87):NE1(1008) *TRP(87):HE1(1016) **VAL(153):O(2413)  *ARG(140):NH2(1868) *ARG(140):1HH2(1873) **PHE(157):O(2467)  *ILE(161):N(2196) *ILE(161):HN(2214) **GLU(14):OE1(193)  *SER(162):N(2215) *SER(162):HN(2224) **GLU(14):OE1(193)  *LYS(172):NZ(2401) *LYS(172):HZ3(2405) **GLU(147):OE1(2312)  *LYS(172):NZ(2401) *LYS(172):HZ3(2405) **GLU(147):OE2(2313)  *GLY(173):N(2406) *GLY(173):HN(2412) **HIS(161):ND1(2542)  *ASN(174):OD1(2424) **HIS(161):HN(2547) **:HIS(161):N(2531) | *ASP(66):CB(691) **VAL(177):CG1(2804)  *TYR(67):CB(703) **VAL(177):CG1(2804)  *TYR(67):CB(703) **VAL(177):CG2(2808)  *TYR(67):CD1(707) **GLY(175):C(2779)  *TYR(67):CE1(711) **GLU(176):CD(2793)  *TYR(67):CE2(713) **LYS(152):CE(2401)  *TYR(67):CE2(713) **GLU(176):CD(2793)  *TYR(67):CZ(715) **GLU(176):CD(2793)  *TRP(70):CD1(755) **LYS(152):CE(2401)  *TRP(70):CE3(759) **GLY(155):CA(2440)  *TRP(70):CE3(759) **GLY(155):C(2443)  *TRP(70):CZ3(764) **GLY(155):C(2443)  *TRP(70):CZ3(764) **GLN(156):CB(2451)  *LEU(71):CD2(784) **GLN(156):CD(2457)  *MET(75):CG(830) **GLN(156):CD(2457)  *TRP(80):CZ3(913) **PHE(157):CD2(2474)  *TRP(87):CZ2(1009) **PRÓ(154):CA(2426)  *TRP(87):CZ2(1009) **PRÓ(154):C(2428)  *TRP(87):CH2(1013) **PRÓ(154):C(2428)  *ARG(140):CB(1856) **PHE(157):CD2(2474)  *ARG(140):CG(1859) **PHE(157):CB(2468)  *ARG(140):CG(1859) **PHE(157):CG(2471)  *ARG(140):CG(1859) **PHE(157):CD2(2474)  *ILE(161):CG2(2206) **THR(103):CG2(1599)  *SER(162):CB(2220) **GLU(14):CD(192)  *LEU(164):CD2(2254) **MET(196):CB(3082)  *LEU(168):CG(2301) **MET(196):CA(3078)  *LEU(168):CD1(2303) **LYS(185):CD(2926)  *LEU(168):CD1(2303) **MET(196):CA(3078)  *LEU(168):CD1(2303) **TRP(197):CD1(3103)  *LEU(168):CD2(2307) **MET(196):CA(3078)  *LEU(168):CD2(2307) **TRP(197):CD1(3103)  *LYS(172):CA(2385) **HIS(161):CE1(2544)  *LYS(172):CD(2395) **HIS(161):CE1(2544) | [*LYS(172):NZ(2401) / *LYS(172):HZ1(2403) / *LYS(172):HZ2(2404) / *LYS(172):HZ3(2405)] -  [**GLU(147):CD(2311) / **GLU(147):OE1(2312) / **GLU(147):OE2(2313)] | - |
| **E** | *ASP(42):OD2(157) **ASN(115):1HD2(1786) **ASN(115):ND2(1783)  *ARG(51):NE(309) *ARG(51):HE(314) **SER(174):O(2768)  *ARG(51):NH2(312) *ARG(51):1HH2(317) **GLY(175):O(2780)  *TRP(70):NE1(639) **LYS(152):HN(2405) **LYS(152):N(2387)  *TRP(70):NE1(639) *TRP(70):HE1(647) **THR(158):O(2487)  *CYS(106):N(1221) *CYS(106):HN(1230) **LYS(163):O(2575)  *SER(147):OG(1877) *SER(147):HG(1879) **ASP(100):OD2(1559) | *TYR(43):CA(160) **PHE(117):CZ(1812)  *TYR(43):C(162) **PHE(117):CZ(1812)  *TYR(43):CB(164) **PHE(117):CZ(1812)  *LEU(47):CD1(238) **PHE(117):CE2(1810)  *LEU(47):CD2(242) **LEU(172):CD1(2741)  *TRP(70):CB(629) **LYS(152):CD(2398)  *TRP(70):CG(632) **LYS(152):CB(2392)  *TRP(70):CD2(635) **LYS(152):CB(2392)  *TRP(70):CD2(635) **LYS(152):CD(2398)  *TRP(70):CE2(636) **LYS(152):CB(2392)  *TRP(70):CE3(637) **LYS(152):CB(2392)  *TRP(70):CE3(637) **LYS(152):CD(2398)  *TRP(70):CZ2(640) **ILE(150):C(2347)  *TRP(70):CZ2(640) **ARG(151):CA(2364)  *TRP(70):CZ2(640) **ARG(151):C(2366)  *TRP(70):CZ3(642) **PHE(180):CE2(2850)  *TRP(70):CH2(644) **ILE(150):C(2347)  *TRP(70):CH2(644) **ILE(150):CG2(2354)  *LEU(71):CD1(658) **THR(160):CG2(2524)  *LEU(71):CD2(662) **THR(158):CB(2488)  *LEU(71):CD2(662) **THR(158):CG2(2490)  *MET(98):CE(1092) **LEU(172):CD1(2741)  *MET(98):CE(1092) **LEU(172):CD2(2745)  *LEU(104):CD2(1209) **LEU(172):CD2(2745)  *ARG(140):CD(1749) **PHE(143):CE2(2235)  *ARG(140):CZ(1753) **PHE(143):CE1(2233)  *ARG(140):CZ(1753) **PHE(143):CZ(2237)  *TYR(144):CD2(1822) **ARG(191):C(3002)  *TYR(144):CD2(1822) **SER(192):CA(3024)  *TYR(144):CE2(1826) **SER(192):CA(3024)  *SER(147):CB(1874) **SER(192):CB(3028)  *ARG(148):CG(1888) **SER(192):CB(3028)  *ARG(148):CD(1891) **SER(192):CB(3028) | - | - |
| **F** | *TRP(87):NE1(993) **PHE(159):HN(2516) **PHE(159):N(2497)  *ARG(90):NE(1054) **HIS(161):HD1(2543) **HIS(161):ND1(2542)  *GLU(94):O(1132) **TRP(197):HE1(3117) **TRP(197):NE1(3109)  *GLU(94):OE2(1141) **LYS(185):HZ3(2936) **LYS(185):NZ(2932)  *ASP(101):OD2(1239) **LYS(194):HZ1(3067) **LYS(194):NZ(3065)  *HIS(102):ND1(1252) *HIS(102):HD1(1253) **LYS(194):NZ(3065)  *GLU(144):N(1929) *GLU(144):HN(1943) **LYS(152):NZ(2404)  *GLU(144):N(1929) *GLU(144):HN(1943) **GLU(176):OE2(2795)  *GLU(144):OE1(1941) **GLN(156):HN(2460) **GLN(156):N(2446)  *LEU(145):N(1944) *LEU(145):HN(1962) **GLU(176):OE2(2795)  *ASP(146):N(1963) *ASP(146):HN(1974) **GLU(176):OE1(2794) | *TRP(80):CD1(889) **GLN(156):CG(2454)  *TRP(80):CD1(889) **GLN(156):CD(2457)  *TRP(80):CE2(892) **GLN(156):CB(2451)  *TRP(80):CZ2(896) **GLN(156):CB(2451)  *TRP(87):CB(983) **THR(158):CG2(2490)  *TRP(87):CD1(987) **PHE(159):CB(2502)  *TRP(87):CD2(989) **THR(158):CA(2484)  *TRP(87):CE2(990) **PHE(159):CD1(2506)  *TRP(87):CE3(991) **THR(158):CG2(2490)  *TRP(87):CZ2(994) **PHE(159):CD1(2506)  *ARG(90):CG(1048) **HIS(161):CE1(2544)  *ARG(90):CD(1051) **HIS(161):CE1(2544)  *GLU(94):CG(1136) **TRP(197):CZ2(3110)  *ILE(95):CG1(1150) **TRP(197):CD1(3103)  *HIS(102):CE1(1254) **GLU(14):CA(182)  *HIS(102):CE1(1254) **GLU(14):C(184)  *HIS(102):CE1(1254) **GLU(14):CB(186)  *HIS(102):CE1(1254) **GLU(14):CG(189)  *PRO(105):CG(1304) **TRP(12):CB(142)  *PRO(105):CD(1307) **TRP(12):CB(142)  *PRO(105):CD(1307) **TRP(12):CG(145)  *THR(138):CA(1833) **THR(160):CG2(2524)  *THR(138):CG2(1839) **THR(160):CG2(2524)  *THR(138):CG2(1839) **LYS(162):CD(2560)  *ARG(139):CZ(1861) **LEU(172):CD1(2741)  *GLY(141):CA(1893) **THR(160):CB(2522) *GLY(141):CA(1893) **THR(160):CG2(2524)  *GLY(141):C(1896) **ILE(150):CG2(2354)  *SER(143):CA(1919) **GLU(176):CD(2793)  *GLU(144):CB(1934) **GLY(155):CA(2440)  *GLU(144):CG(1937) **LYS(152):CB(2392)  *GLU(144):CG(1937) **LYS(152):CG(2395)  *LEU(145):CA(1945) **GLU(176):CD(2793)  *LEU(145):CB(1949) **GLU(176):CG(2790)  *LEU(145):CB(1949) **GLU(176):CD(2793)  *LEU(145):CG(1952) **GLU(176):CG(2790)  *LEU(145):CG(1952) **GLU(176):CD(2793)  *LEU(145):CD1(1954) **GLU(176):CG(2790)  *LEU(145):CD2(1958) **PRO(154):CB(2430) | [*HIS(102):CG(1249) / *HIS(102):CD2(1250) / *HIS(102):ND1(1252) / *HIS(102):HD1(1253) / *HIS(102):CE1(1254) / *HIS(102):NE2(1256) / *HIS(102):HE2(1258)]  [**GLU(14):CD(192) / **GLU(14):OE1(193) / **GLU(14):OE2(194)]    [*GLU(64):CD(655) /*GLU(64):OE1(656) / *GLU(64):OE2(657)] - [**HIS(23):CG(343) / **HIS(23):CD2(344) / **HIS(23):ND1(346) / **HIS(23):HD1(347) / **HIS(23):CE1(348) / **HIS(23):NE2(350) / B:HIS(23):HE2(352)]  [*ASP(135):CG(1807) / *ASP(135):OD1(1808) / *ASP(135):OD2(1809)] - [**LYS(162):NZ(2566) / **LYS(162):HZ1(2568) / **LYS(162):HZ2(2569) / **LYS(162):HZ3(2570)]  [*GLU(94):CD(1139) / *GLU(94):OE1(1140) / *GLU(94):OE2(1141)] - [**LYS(185):NZ(2932) / **LYS(185):HZ1(2934) / **LYS(185):HZ2(2935) / **LYS(185):HZ3(2936)]  [*ASP(101):CG(1237) / *ASP(101):OD1(1238) / *ASP(101):OD2(1239)] - [**LYS(194):NZ(3065) / **LYS(194):HZ1(3067) / **LYS(194):HZ2(3068) / **LYS(194):HZ3(3069)] | [*TYR(104):CG(1283) / *TYR(104):CD1(1284) / *TYR(104):CE1(1288) / *TYR(104):CZ(1292) / *TYR(104):CE2(1290) / *TYR(104):CD2(1286)] - [**:TRP(12):CE2(149) / **TRP(12):CD2(148) / **TRP(12):CE3(150) / **TRP(12):CZ3(155) / **TRP(12):CH2(157) / **TRP(12):CZ2(153)] |
| **H** | *ARG(51):NH2(284) *ARG(51):1HH2(289) **GLU(176):OE2(2795)  *ARG(51):NH2(284) **VAL(177):HN(2812) **VAL(177):N(2797)  *ARG(51):NH2(284) *ARG(51):2HH2(290) **VAL(177):O(2801)  *HIS(55):NE2(356) *HIS(55):HE2(358) **PRÓ(154):O(2429)  *GLN(62):OE1(481) **GLN(156):2HE2(2462) **GLN(156):NE2(2458)  *ASP(74):O(665) **PHE(159):HN(2516) **PHE(159):N(2497)  *HIS(75):NE2(688) **HIS(161):HD1(2543) **HIS(161):ND1(2542)  *TYR(95):OH(1015) *TYR(95):HH(1017) **THR(160):OG1(2528) | *LYS(35):CA(3) **LEU(172):CD1(2741)  *LYS(35):CG(10) **LEU(172):CB(2736)  *LYS(35):CE(16) **THR(173):C(2753)  *TYR(43):CD2(142) **GLY(175):CA(2776)  *TYR(43):CE2(146) **GLY(175):CA(2776)  *LEU(47):CD1(210) **GLY(175):C(2779)  *LEU(47):CD1(210) **GLU(176):CA(2783)  *LEU(47):CD1(210) **GLU(176):CG(2790)  *LEU(47):CD1(210) **GLU(176):CD(2793)  *LEU(47):CD2(214) **GLU(176):CD(2793)  *PHE(54):CE2(336) **GLY(155):C(2443)  *PHE(54):CE2(336) **GLN(156):CB(2451)  *PHE(54):CZ(338) **GLN(156):CB(2451)  *PHE(54):CZ(338) **THR(158):CG2(2490)  *LEU(61):CB(454) **GLN(156):CD(2457)  *LEU(61):CD1(459) **GLN(156):CG(2454)  *GLY(73):CA(655) **PHE(159):CE1(2510)  *GLY(73):CA(655) **PHE(159):CZ(2514)  *GLY(73):C(658) **PHE(159):CD1(2506)  *GLY(73):C(658) **PHE(159):CE1(2510)  *ASP(74):CA(662) **PHE(159):CB(2502)  *ASP(74):CA(662) **:PHE(159):CG(2505)  *ASP(74):C(664) **PHE(159):CB(2502)  *HIS(75):CD2(682) **HIS(161):CE1(2544)  *VAL(76):CB(696) **THR(158):CG2(2490)  *VAL(76):CG1(698) **THR(158):CG2(2490)  *TYR(95):CD2(1008) **THR(160):CG2(2524)  *TYR(95):CE2(1012) **THR(160):CG2(2524)  *TYR(95):CZ(1014) **THR(160):CG2(2524)  *MET(98):CA(1053) **LEU(172):CD1(2741)  *GLN(100):C(1096) **LEU(172):CD2(2745) | [*ASP(74):CG(669) / *ASP(74):OD1(670) / *ASP(74):OD2(671)] - [**HIS(161):CG(2539) / **HIS(161):CD2(2540) / **HIS(161):ND1(2542) / **HIS(161):HD1(2543) / **HIS(161):CE1(2544) / **HIS(161):NE2(2546) / **HIS(161):HE2(2548)]  [*ARG(51):CZ(282) / *ARG(51):NH1(283) / *ARG(51):NH2(284) / *ARG(51):1HH1(287) / *ARG(51):2HH1(288) / *ARG(51):1HH2(289) / *ARG(51):2HH2(290)] -  [**GLU(176):CD(2793) / **GLU(176):OE1(2794) / **GLU(176):OE2(2795)] |  |

Isoform * | receptor ** | Hydrogen bonds (HB) | Hydrophobic contacts (HC) | Salt bridges (SB) | T-shaped
