# Supplementary material for: Molecular modelling of the HCMV IL-10 protein isoforms and analysis of their interaction with the human IL-10 receptor
Source: PLoS One. 2022 Nov 28;17(11):e0277953. doi: 10.1371/journal.pone.0277953 (PMC9704672; doi:10.1371/journal.pone.0277953)
Supplement: S1 Raw images — (PDF) [file pone.0277953.s009.pdf]

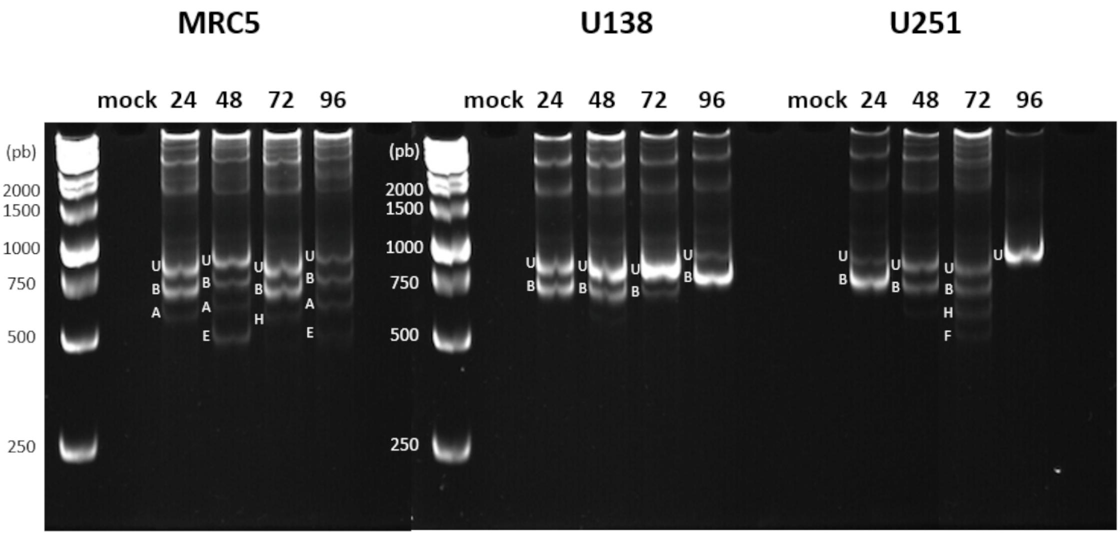

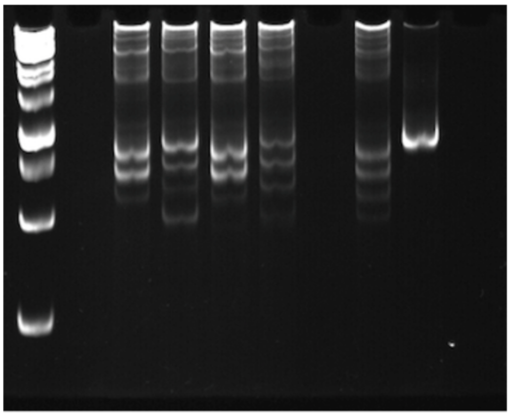

Image containing respectively ladder, mock MRC5, 24, 48, 72 and 96 hpi. It also contains U251 72 and 96 hpi.

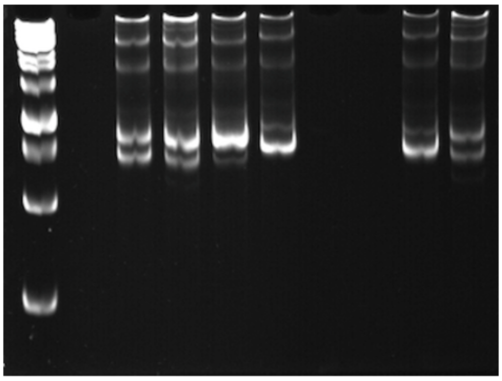

Image containing respectively ladder, mock U138, 24, 48, 72 and 96 hpi. Also contains U251 mock, 24, and 48 hpi.
