# Supplementary material for: Molecular modelling of the HCMV IL-10 protein isoforms and analysis of their interaction with the human IL-10 receptor
Source: PLoS One. 2022 Nov 28;17(11):e0277953. doi: 10.1371/journal.pone.0277953 (PMC9704672; doi:10.1371/journal.pone.0277953)
Supplement: Attachment — Submitted filename: Rebutal Letter.docx [file pone.0277953.s011.docx]

To PlosONE

Manuscript  #: PONE-D-22-17017

Dear reviewers

We thank very much for the revision of the manuscript. We are very pleased about the revision. Bellow are the responses for the specific comments.

**General corrections**

-The manuscript was revised to match the PLOS ONE’s style requirements. Including the files names.

-We included, in the financial disclosure, the following sentence. “The founders had no role in study design, data collection and analysis, decision to publish, or preparation of the manuscript”.

-The Data availability statement has been modified. The data are available in https://www.kaggle.com/datasets/tainanneves/data-availability-hcmv-il10-molecular-models.

-We added the sequence identifier numbers of genbank in the manuscript.

-The Orcid ID was associated with the corresponding author of the manuscript in the PLOS ONE system.

-The gel and blot original images are available in the supplementary materials as a PDF file.

-Legends of the supplementary materials are available in the final of the manuscript.

-The bibliography has been revised

**Reviewer's Responses to Questions**

responses to the specific comments.

**Reviewer #1:** 

- Text describing experiments shown in Figs 4&5 would be improved by more clearly describing the rationale behind focusing on isoform F.

Response: We better described the focus in isoform F in the Results / Molecular docking section and Discussion. This isoform likely stablish the most stable complex having a consistent increasing in its RMSF number when in its complexed conformation. One can imagine that the ability to bind with higher affinity to the receptor could result in enhanced signalling or in competitive inhibition of binding by other isoforms to IL-10R1, therefore we focused on isoform F.

- Increased stability of Isoform B with IL-10R1 versus Isoform A is very curious. The discussion of this finding by the authors is good, however I think more explanation as to why Isoform A could still be more biologically active would improve the manuscript. What evidence or precedent is there that suggests that Isoform A intersection of binding sites 1 and 2 would enhance biological activity?

Response: As far as we know there is no precendent evidence suggesting that intersection of binding sites 1 and 2 enhances the biological activity of isoform A. According to our data we suggest that **this may occur.** We can observe that isoform sites 1 and 2 in isoform A are very close to each other, possibly forming a large site, favouring connections and improving the binding possibilities.

We describe it better throughout the text, mainly in the results section.

- The manuscript could be improved overall by some editing, with paragraphs needing editing (e.g., removal/editing of very short paragraphs).

Response: We performed a removal of short paragraphs and textual corrections.

Minor comments

- Line 315 Fig.2. Structure and aminoacid – needs a space

Corrected

- 321 – gray > grey

Corrected

- 552 - Therefore, despite of exhibiting – should read ‘in spite of’

Corrected

**Reviewer #2:**

The impact of the study is somewhat limited due to the in-silico approach used to assess potential cmvIL-10 isoform interaction with IL-10R1.

Also, the authors suggest strain variation or PCR technique as possible explanations for the absence of cmvIL-10 isoform H in previous publications.

It would be interesting to see the authors perform their transcriptional and sequencing analysis approach on cells infected with AD169 to directly address this issue, although I don't think it is a requirement for publication.


Minor issues:
1. The Y-axis label in Fig 5A is clipped

Corrected
